# Bioactive Compounds and Their Derivatives: An Insight into Prospective Phytotherapeutic Approach against Alzheimer's Disease

**DOI:** 10.1155/2022/5100904

**Published:** 2022-04-11

**Authors:** Fahadul Islam, Jannatul Fardous Khadija, Md. Harun-Or-Rashid, Md. Saidur Rahaman, Mohamed H. Nafady, Md. Rezaul Islam, Aklima Akter, Talha Bin Emran, Polrat Wilairatana, Mohammad S. Mubarak

**Affiliations:** ^1^Department of Pharmacy, Faculty of Allied Health Sciences, Daffodil International University, Dhaka 1207, Bangladesh; ^2^Faculty of Applied Health Science Technology, Misr University for Science and Technology, Giza 12568, Egypt; ^3^Department of Pharmacy, BGC Trust University Bangladesh, Chittagong 4381, Bangladesh; ^4^Department of Clinical Tropical Medicine, Faculty of Tropical Medicine, Mahidol University, Bangkok, Thailand; ^5^Department of Chemistry, The University of Jordan, Amman 11942, Jordan

## Abstract

Alzheimer's disease (AD) is a common neurodegenerative brain disorder that causes cellular response alterations, such as impaired cholinergic mechanism, amyloid-beta (A*β*) AD aggregation, neuroinflammation, and several other pathways. AD is still the most prevalent form of dementia and affects many individuals across the globe. The exact cause of the disorder is obscure. There are yet no effective medications for halting, preventing, or curing AD's progress. Plenty of natural products are isolated from several sources and analyzed in preclinical and clinical settings for neuroprotective effects in preventing and treating AD. In addition, natural products and their derivatives have been promising in treating and preventing AD. Natural bioactive compounds play an active modulatory role in the pathological molecular mechanisms of AD development. This review focuses on natural products from plant sources and their derivatives that have demonstrated neuroprotective activities and maybe promising to treat and prevent AD. In addition, this article summarizes the literature pertaining to natural products as agents in the treatment of AD. Rapid metabolism, nonspecific targeting, low solubility, lack of BBB permeability, and limited bioavailability are shortcomings of most bioactive molecules in treating AD. We can use nanotechnology and nanocarriers based on different types of approaches.

## 1. Introduction

Alzheimer's disease (AD) is a progressive neurological disorder that affects memory and cognitive function as individuals age [1–3]. It accounts for 60-80% of all dementia cases, ranking it the fifth leading cause of mortality [4]. As a neurodegenerative disorder (NDD), AD steadily and permanently diminishes memory, cognition, and ultimately the capability of regular activities, necessitating full-time care. The disease is most prevalent in those over 65 but can also harm younger people. One of the most significant risk factors for AD is age. Research findings showed that the percentage of people with Alzheimer's dementia increases dramatically with age, where 3% of people aged 65-74, 17% of people aged 75-84, and 32% of people aged 85 or older have AD dementia [5, 6]. The pathogenesis of AD is affected by both environmental and genetic factors [7]. In this respect, extracellular depositing in amyloid and intracellular neurofibrillary tangles (NFTs) are two prominent pathologic hallmarks of AD [8]. Accumulation of amyloid causes cognitive decline, leading to clinical dementia [9]. Both amyloid precursor protein (APP) and presenilin mutations end up with different production of amyloid-beta (A*β*) peptides and neuronal death, which is involved in AD development [10, 11]. Surprisingly, new data indicated that neuroinflammation is a critical pathological constituent of Alzheimer's [12]. During AD pathogenesis, the extracellular protein A*β* aggregates, whereas intracellular neurofibrillary tangles (NFTs) reflect hyperphosphorylation of tau proteins in neurons. These neurons generate neuronal cell death and are primarily found in the brain's cerebral cortex and hippocampus [13]. Deposition of aggregated A*β* protein in the synapses of Alzheimer's patients causes inflammation and oxidative stress (OS). In addition, AD consists of excessive glutamatergic neurotransmission and cholinergic neurotransmission depletion [14].

The most frequent and early symptom of AD dementia is the inability to recall memories [15]. Immediate memory impairment frequently occurs, but distant memory is also impacted later, and short-term memory impairment is generally the first clinical sign. On the other hand, memory processing that does not require hippocampal structures is not impaired in AD [16, 17]. In *late-stage severe* dementia due to AD, people generally lose the ability to move and communicate coherently, leading to substantial memory loss and the loss of their sense of time and place. In this case, patients require additional care. Over the next 50 years, it is anticipated that therapeutic intervention that can delay the onset or progression of AD would dramatically reduce the number of cases [18]. Natural products and their bioactive molecules have been widely shown as successful and promising sources of potential drug leads in the development of medications for the treatment of AD [19, 20]. In this respect, mixtures or extracts of natural products contain natural bioactive molecules that could be a potential therapeutic strategy for treating or preventing AD [21–23]. Furthermore, several extracts and natural sources are broadly used in animal models and clinical trials to cure AD [24, 25]. Based on the previous discussion, and owing to the wide range of preventive and therapeutic options of natural products of plant origin, this review highlights the therapeutic ability of plant-based natural products that may have neuroprotective properties for the control and treatment of AD through various mechanisms.

## 2. Methodology

**Figure 1:**
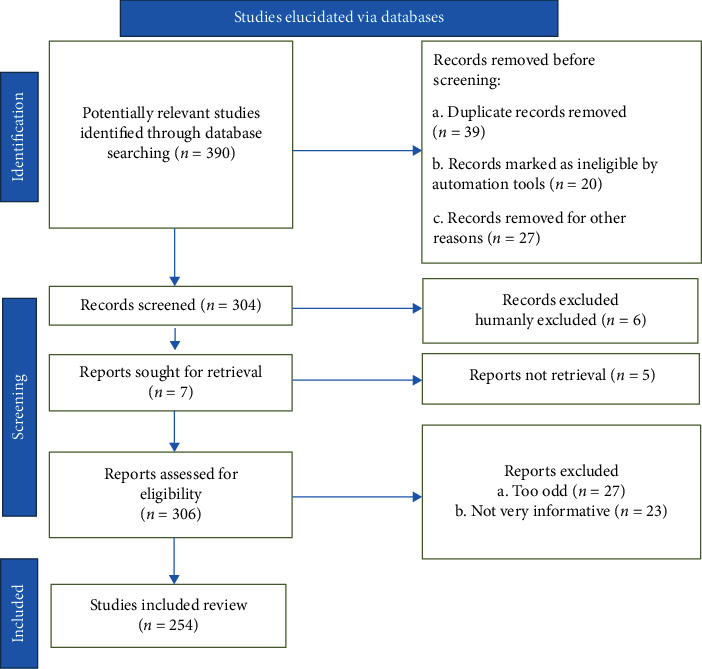
Stages involved in selecting published data for inclusion in the present study are depicted in a flow chart; *n*: number of literature reports.

We performed a literature search, and recent relevant references have been obtained from different databases such as Scopus, Science Direct, Elsevier, PubMed, and Web of Science. In our search, we used the following terms: medicinal plant, neuroprotection, Alzheimer's disease, antioxidant, and inflammation. Research reports, review articles, and original research articles in English published up to October 2021 were selected and evaluated. In addition, we examined the citations therein and included them when appropriate. According to the recommendations of Page et al. [26, 27], an algorithm was used that inserted all of the steps involved for selecting the relevant information in the study, as indicated in the flow chart in Figure 1. We reviewed 390 references and included 254 of them in the present review.

## 3. Pathology of AD

Amyloid plaques and NFTs are the pathological hallmarks of AD. In addition, neuropil threads, dystrophic neurites, related astrogliosis, microglial activation, and cerebral amyloid angiopathy are also seen in AD [28]. Neurodegeneration, including synaptic and neuronal loss, leads to macroscopic atrophy due to these pathologic processes. Mixed pathology, which comprises vascular disease and Lewy bodies, is also common neurodegenerative dementia in older people [29]. Indeed, Lewy body pathology frequently coexists with familial AD, the mechanism for which is unknown [30]. Moreover, TDP-43 pathology is becoming more acknowledged as a pathology [31].

Amyloid plaques are extracellular accumulations primarily made up of improperly folded A*β* proteins with 40 or 42 amino acids (A*β*40 and A*β*42), two by-products of APP metabolism. Because of its increased rate of insolubility and fibrillation, A*β*42 is more common in plaques than A*β*40. Amyloid deposition may not necessarily comply with a predictable development sequence, but it generally begins in the isocortex and only affects subcortical regions later. Unlike NFTs, amyloid plaques have a minor impact on the entorhinal cortex and hippocampal formations. On the other hand, hyperphosphorylated tau paired helical filaments (PHFs) make up the majority of NFTs. Tau disease frequently starts in the medial temporal lobes (entorhinal cortex and hippocampus) and then spreads to the associative isocortex; the primary sensory, motor, and visual domains are mostly freed. Because neuronal and synapse loss often coincides with tangle development, pathology of NFT is better connected with clinical characteristics and severity of AD [32]. A*β* pathology, on the other hand, achieves a plateau early in the disease's clinical phase [33].

## 4. Etiology and Pathophysiology of AD

Although Aloise Alzheimer, a German doctor, initially described AD over a century ago, the fundamental mechanisms responsible for its development are still unknown [34]. Dementia, memory loss, mobility dysfunction, sadness, delusion, spatial awareness impairment, and hallucination are common AD signs, and anomic aphasia, acalculia, and apathy are common symptoms. Furthermore, patients in the terminal stages of the disease cannot speak vocally, have lost their independence, and cannot conduct basic daily tasks [35, 36]. These behavioral abnormalities observed in AD are symptoms of the underlying CNS processes. Despite ongoing research, the etiopathogenesis of this condition has yet to be fully understood. However, some distinct pathways have been found at the cellular and tissue levels. In this context, the buildup of A*β* is a common symptom of AD. A*β* is a short peptide synthesized from naturally existing APP and produces senile plaque APP. Furthermore, A*β* regulates synaptic plasticity, has a role in axonal growth, and modulates axonal growth under physiological settings [36, 37].

**Figure 2:**
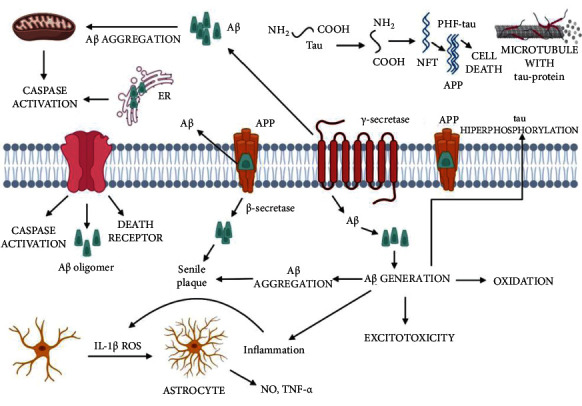
Amyloid cascade in Alzheimer's disease (AD). ER: endoplasmic reticulum; A*β*: amyloid-beta; NFT: neurofibrillary tangle; APP: amyloid precursor protein, ROS: reactive oxygen species; NO: nitric oxide; TNF-*α*: tumor necrosis factor-alpha [38].

Other pathological alterations that disrupt the structure of pyramidal neurons often accompany development. Increased phosphorylation of tau proteins causes these processes, resulting in tau tangles's formation (Figure 2). In addition, tau proteins are involved in the microtubule's stabilization and, therefore, the cytoskeleton structure in physiological settings. In this respect, microtubules provide a proper neuron function and synaptic signaling by acting as an enzyme transporter and cellular protein. Microtubules are delicate structures that rely on interactions between their fundamental ingredients, tubulins, and tau proteins, for stability. Increased tau phosphorylation induces microtubule disassembly and increases the production of tau tangles, which is characteristic of AD. Similarly, the increased concentration of Ca^2+^ ions caused directly through the aggregation of A*β* inside nerve cells can trigger cyclin-dependent kinase five as one of the agents implicated in this process. Thus, microtubules depolymerize, the cytoskeleton deforms, intracellular transport is disrupted, and the neuron's overall function is reduced. Toxic aggregates represented in tau tangles activate microglia, causing inflammation, neuronal damage, and cell death to combine into toxic aggregates.

Although several hypotheses have been proposed to explain the pathology of AD, the exact mechanism remains unknown and complex [39]. The following are a few of the hypotheses that have been proposed:

### 4.1. Cholinergic Hypothesis

Mental state, brain adaptation, sleep-wake cycle regulation, cerebral blood flow control, and neuronal function are affected by cholinergic neurotransmission. According to investigations, the cholinergic system is also essential in cognitive performance. Therefore, impairment could result in memory loss [40, 41]. During cholinergic neurotransmission, acetylcholine (ACh) is released within synapses. Hydrolysis of ACh, which results in signal termination, is carried out by acetylcholinesterase (AChE) and butyrylcholinesterase (BuChE). In this context, certain AD patients showed unaltered or enhanced BuChE activity. AChE is also linked to the development of neurotoxic A*β* fibrils, implying that AChE-induced A*β* aggregation contributes to the advancement of AD, which leads to AChE and BuChE inhibition as a hopeful technique for treating AD [42].

### 4.2. Hypothesis of Amyloid

A*β* precursor protein (APP) is encoded by a single gene on chromosome 21's 19 exons and considers a type I transmembrane sialoglycoprotein. There are three isoforms of APP: APP751, APP770, and APP695. Neurite outgrowth, cell adhesion, intracellular calcium level stabilization, and synaptic plasticity regulation are APP functions. APP in its soluble form exhibits neurotrophic and neuroprotective effects [43]. There are two types of APP processing pathways: nonamyloidogenic and amyloidogenic. The significant process involves cleavage of Lys16 of the APP by *α*-secretase enzyme, which results in an A*β* soluble peptide and C-terminal fragment. On the other hand, the nonamyloidogenic peptide p3 is produced by dividing the C-terminal portion by *γ*-secretase. In contrast, *β*-secretase cleaves APP to create soluble A*β* peptides and a C-terminal segment. Furthermore, *γ*-secretase cleaves APP at numerous locations resulting in an A*β* monomer containing amino acids in the 38–43 range. Then, A*β* monomers self-assemble into neurotoxic oligomers; subsequently, the creation of fibrillary aggregates causes neuronal malfunction, which eventually leads to dementia [44]. Aggregated oligomers also cause the production of senile plaques, which are a hallmark of AD. In Alzheimer's patients, levels of the A*β*_42_ peptide are relatively higher than usual. Furthermore, genetic studies show that APOE, PSEN1, and PSEN2 affect A*β* pathogenesis [45].

### 4.3. Hypothesis of Tau

Tau protein is a phosphoprotein with 38 phosphorylation sites and six isoforms which vary in length from 352 to 441 amino acids. In this respect, the domains of tau are defined based on their microtubule interactions and their amino acid character. In this case, the amino-terminal fragment does not bind to microtubules but instead projects away from the microtubule surface is termed “projection domain.” Furthermore, the projection domain is separated into amino-terminal and proline-rich regions rich in acidic residues. Likewise, there is division of the tubulin-binding part and the acidic carboxy-terminal portion of the microtubule-binding domain [39]. Phosphorylated tau protein helps stabilize axonal microtubule assembly and participates in intracellular trafficking by interacting with tubulin [46]. Due to aberrant tau phosphorylation, normal tau is transferred to NFTs and paired helical filament tau (PHF-tau). Hyperphosphorylated tau destabilizes microtubules, resulting in nerve cell death. Findings indicated that hyperphosphorylated tau in an AD patient's brain is three to four times higher than in the normal brain [47].

### 4.4. Neuroinflammation

Elevated levels of microglia and astrocytes cause chronic neuroinflammation by releasing proinflammatory cytokines such as interferon, interleukin-1, and tumor necrosis factor (TNF), which have been found in AD patients and affect the brain. In this regard, reactive oxygen species (ROS) enhance the effect of *β*-secretase, which cleaves APP to generate an A*β* peptide [46]. Consequently, anti-inflammatory techniques have been employed to produce new compounds that can be used to treat and prevent AD [48].

### 4.5. Biometal Dyshomeostasis

Metals such as copper, iron, and zinc are essential in biologically essential activities such as protein structure stability, metabolism, catalyst activity, and cellular signal communication [49]. Increased DNA, proteins, and lipids may be produced by free radical generation by the Fenton reaction, primarily powered by redox-active Fe^2+^ and Cu^2+^. Therefore, in neurodegenerative disorders such as AD, dysregulation of biometals leads to an increase in oxidative stress, which is why metal chelators could have a role in breaking AD development [50, 51].

### 4.6. Oxidative Stress (OS)

Consumption of oxygen and cellular signaling produce ROS such as hydroxyl radical, superoxide anion radical, peroxide, and hydrogen peroxide. The intrinsic antioxidant system regulates ROS balance in normal conditions [52]. However, there is a discrepancy between ROS generation and clearance in pathological situations, leading to high ROS levels [53]. Research findings indicated that brain OS may be an early event in AD and might affect disease development [54, 55]. The brain is the most energy-demanding organ, consumes more oxygen than other organs, and conducts mitochondrial respiration, thus increasing the risk of exposure to ROS. On the other hand, protein oxidation and lipid peroxidation play a role in forming and deposition of A*β* in AD [56, 57].

### 4.7. Insulin-Degrading Enzyme

AD is linked to type 2 diabetes and insulin resistance in the brain. Studies have associated the insulin-degrading enzyme (IDE) to the deposition of A*β* and tau hyperphosphorylation. Insulin and A*β* consider IDE as a competing substrate that plays a role in the pathophysiology of AD. In addition, clearing of A*β* in the brain is linked to IDE. Consequently, IDE activators can be used to treat AD.

### 4.8. Homocysteine

After demethylation of methionine, homocysteine (HCy) is a nonproteinogenic homolog of cysteine. HCy binds to glutamate NMDA receptors, causing glutamate excitotoxicity, which leads to neurotoxicity and, eventually, neuronal death. Oxidative damage, apoptosis, A*β* aggregation, and tau protein hyperphosphorylation are linked to high levels of HCy [58, 59].

### 4.9. Phosphodiesterase

Phosphodiesterases (PDEs) are enzymes that help to break down cyclic guanosine monophosphate (cGMP) and cyclic adenosine monophosphate (cAMP). Moreover, they play a role in synaptic plasticity and intracellular signaling cascade control. Changes in PDE4, PDE7, and PDE8 expression, in particular, have been linked to AD [60].

## 5. Natural Products

**Figure 3:**
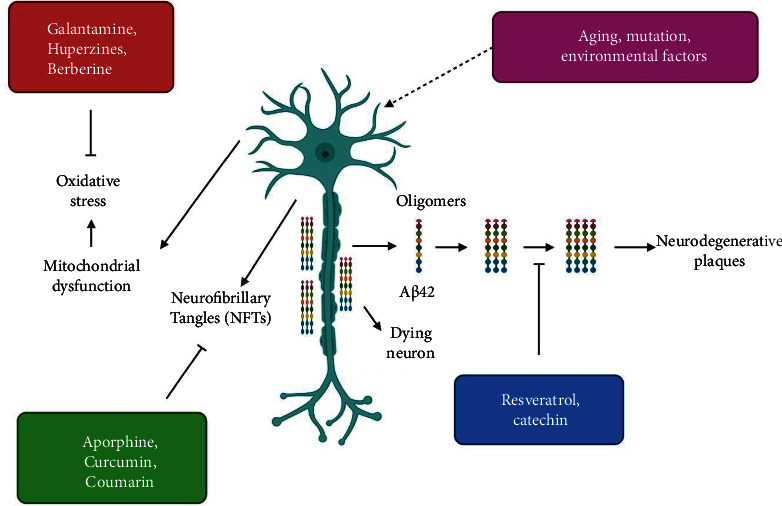
Illustration of the mode of actions by which natural products block Alzheimer's disease (AD).

Research findings indicated that some dietary components lessen the incidence of AD, prompting scientists to investigate the activity of plant bioactive molecules [61]. Natural bioactive molecules are regarded as “secondary metabolites” of plants. In this respect, numerous chemicals extracted from several plants, including roots, rhizomes, leaves, and seeds, have been shown to inhibit harmful plaque development and increase cholinergic signaling [62, 63] (Figure 3).

**Figure 4:**
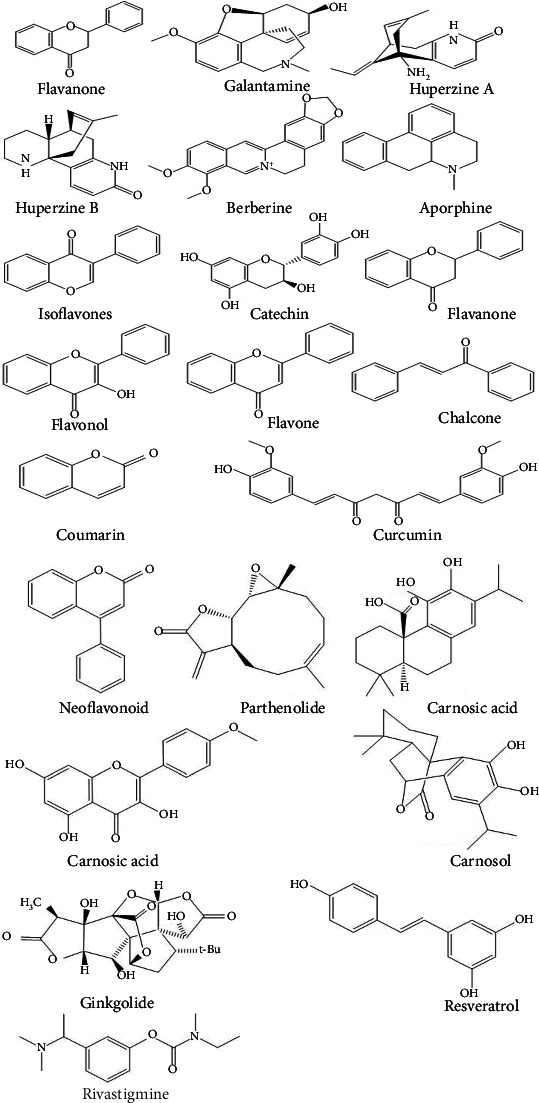
Chemical structures of some chemical compounds that are effective against Alzheimer's disease (AD).

Antioxidant-rich foods lower oxidative stress in the brain. Accordingly, plant-derived products exhibit a wide range of pharmacological effects and attract the attention of scientists who want to use them to develop molecules to cure various ailments [64, 65]. Findings showed that some natural bioactive compounds are adequate for AD management. Below are details about these compounds (Figure 4).

### 5.1. Alkaloids

Alkaloids, a family of nitrogenous chemical substances, are abundant in confirmed flowering plant families. Many species contain a limited number of alkaloids, but others, such as the Solanaceae, Papaveraceae, Amaryllidaceae, and Ranunculaceae, have many alkaloids [62, 66]. In addition, amphibians include the poison dart frog, rodents like the new world beaver, and fungi like ergot produce alkaloids. Interestingly, rivastigmine and galantamine, two AChEIs approved by the Food and Drug Administration (FDA) in the United States, are alkaloids [64, 66].

#### 5.1.1. Galantamine

Galantamine is an isoquinoline alkaloid found in the flowers and bulbs of *Galanthus caucasicus*, *Galanthus sworonowii*, and *Leucojum aestivum*, all of which are members of the Amaryllidaceae family [67]. Galantamine functions as an allosteric modulator of nicotinic acetylcholine receptors (nAChRs) [68, 69]. Several galantamine derivatives were synthesized by connecting them to memantine with linkers of various lengths and compositions. These compounds were tested as AChE inhibitors and for selectivity toward NMDAR binders and NMDAR subunit 2B (NR2B). Some synthesized compounds showed outstanding inhibitory activity against AChE where IC_50_ was nanomolar and micromolar affinities for NMDAR. When tested for selectivity toward NMDAR containing the 2B subunit (NR2B), some derivatives exhibited a micromolar affinity for NR2B. Finally, selected compounds were tested using a cell-based assay to measure their neuroprotective activity. In addition, three of the prepared compounds exhibited showed remarkable neuroprotective activities, inhibiting the NMDA-induced neurotoxicity at subnanomolar concentrations, and the IC_50_ value of one of the prepared compounds was 0.28 nM [70].

In addition, a new dual-site binding hybrid comprising galantamine and indole was synthesized and docked on rhAChE. Results revealed AChE inhibitory activity with IC_50_ values of 0.011 *μ*M, 0.012 *μ*M, and 0.015 *μ*M for three of the synthesized compounds. The galantamine moiety interacts with the CAS, while the indole portion interacts with the aromatic residues in the peripheral anionic site, resulting in galantamine-indole derivatives that act as dual site binders to the rhAChE enzyme [71].

#### 5.1.2. Huperzines

Huperzines A and B, two lycopodium alkaloids, are isolated from *Huperzia serrata* (club moss), a Chinese medicinal herb, which can treat illnesses such as swelling, confusion, schizophrenia, fever, and strain. Huperzine A inhibits AChE and BuChE in a specific, effective, and reversible manner, with IC_50_ values of 0.82 and 74.43 nM, respectively [72, 73]. Similarly, huperzine B is a reversible inhibitor of AChE with an IC_50_ of 14.3 *μ*M [74]. Consequently, huperzines A and B are frequently employed as natural moieties in developing more powerful AChEIs. In addition, novel AChE inhibitors based on huperzine A's carbobicyclo and tacrine's 4-aminoquinoline substructures with several substituents have been presented as possible AChE inhibitors [75]. Moreover, various heterodimers containing donepezil dimethoxyindanone and huperzine A pyridone connected via a different methylene linker have been considered AChE inhibitors with possible importance in treating AD [76]. Interestingly, novel huperzine A and imine derivatives containing an extra tiny substituted aromatic ring demonstrate efficiency in the nanomolar range, as hAChE inhibitors, where the huperzine derivatives' aromatic rings reveal a *π*-*π* stacking with AChE amino acid residues [77].

Furthermore, novel huperzine B compounds were wisely developed. The huperzine B moiety is attached to the terminal aromatic ring by a tether chain, favoring interaction with the peripheral anionic site (PAS) [78]. The hydroxyanthraquinone system of rhein has been connected to a unit of heparin Y using a variety of linkers to create novel multitarget rhein-huprin hybrids. Huprin Y interacts with an active catalytic site (CAS), whereas rhein's aromatic rings form *π*-*π* stacking contacts with AChE's PAS, resulting in a dual-site inhibitor [79].

#### 5.1.3. Berberine

Berberine is a benzylisoquinoline alkaloid isolated from rhizomes, stems, roots, and bark of *Berberis* spp. and *Phellodendron amurense*. It exhibits potent antioxidant, anti-inflammatory, anticancer, antibacterial, cardioprotective, and neuroprotective activities [80–82]. In addition, berberine inhibits both AChE and BuChE; however, AChE inhibition is more selective. Berberine also inhibits the voltage-dependent potassium current and exhibits an antagonistic effect against the NMDA receptor (especially NR1), resulting in neuroprotection. As a result, berberine improves cognitive impairment in AD via increasing cholinergic stimulation [83, 84]. Furthermore, *Torpedo californica* acetylcholinesterase (TcAChE) was docked with novel dual-site binding derivatives of triazole and berberine moieties [85]. Berberine-thiophenyl hybrids were synthesized by replacing the oxygen or NH group of the berberine derivatives with a sulfur atom, which caused increasing antioxidant properties. These hybrids also showed antioxidant effects and prevented A*β* aggregation [86].

#### 5.1.4. Aporphine

Aporphine alkaloids are part of the alkaloid isoquinoline class and feature a tetrahydroisoquinoline substructure and are isolated from *Menispermum dauricum* [87, 88]. Aporphine alkaloids such as oxoisoaporphine and oxoaporphine exert numerous biological activities, including the ability to inhibit telomerase cholinesterase and A*β* aggregation and antioxidant activity [87, 89]. In this regard, synthetic oxoaporphine derivatives are two to three times less potent than their oxoisoaporphine analogs as AChE inhibitors. According to molecular modeling studies, the azabenzanthrone moiety of the oxoisoaporphine alkaloids could attach to Trp279 residue of PAS of AChE via *π*-*π* stacking interaction. Using amines or ammonium groups as spacers considerably improved the water solubility and selectivity of the oxoisoaporphine alkaloid toward AChE [87]. An aminoalkyl tether connected a new set of oxoisoaporphine-tacrine hybrids. These novel compounds exhibited antiaggregating activity; they were potent inhibitors of self-induced A*β* aggregation at 10 *μ*M concentrations (35.5–85.8%) [89]. Moreover, dealkylation and ring aromatization processes created eight nuciferine derivatives. AChE inhibitors with IC_50_ values of 28 and 25 *μ*g/mL were discovered in 1,2-dihydroxyaporphine and dehydronuciferine products [90].

### 5.2. Flavonoids and Other Polyphenols

Flavonoids are polyphenols found in fruits and vegetables. These are abundant in Polygonaceae, Rutaceae, and Leguminosae plant families [47, 64]. Flavonoids exhibit neuroprotective activities because of their polyphenolic composition; they scavenge free radicals such as superoxide radicals and hydrogen peroxide. The frequency and position of hydroxyl groups in polyphenols influence their capacity to scavenge free radicals. Because of their antioxidant characteristics, a new line of flavonoid derivatives was synthesized [65, 66]. The position of the B ring, the degree of unsaturation, and oxidation of the C ring split flavonoids into various subgroups. Isoflavones are compounds with the B ring connected to position 3 of the C ring, resulting in the 3-phenylchromen-4-one structure. In contrast, in neoflavonoids, the B ring is attached to position 4 of the C ring, resulting in a 4-phenylcoumarine structure. The B ring is connected to position 2 of the C ring in the following subgroups: flavones, flavonols, flavanones, flavan-3-ol or flavanols or catechins, and chalcones; only the structural properties of the C ring differ [91].

Furthermore, flavonoids have become widely employed phytochemicals with different medicinal effects. Due to their core repressive activities against proinflammatory transcription factors, flavonoids play a central role in reducing neuroinflammation in AD [92]. In addition, this category of substances activates transcription factors of antioxidant and anti-inflammatory. Though flavonoids have the potential to be a natural treatment in preclinical AD models, it is, however, worth mentioning that the parent flavonoids' average bioavailability is frequently low. Flavonoids also pass the blood-brain barrier (BBB) because of their intense polarization [12].

#### 5.2.1. Flavones

Flavones are frequently found in numerous medicinal plants, providing several health benefits. Flavones and derivatives inhibit advanced glycation products (AGEs) and exhibit biological activities such as antioxidant, anti-inflammatory, and neuroprotective. These compounds could also be promising agents for treating and preventing AD [92–100].

#### 5.2.2. Isoflavones

Isoflavonoids can be obtained in leguminous plants, such as soybeans, and extracted from microorganisms. During plant-microbe interactions, they function as precursors for the formation of phytoalexin. These compounds exert an inhibitory action on AChE and MAO-B [101].

#### 5.2.3. Flavanones

Another significant subgroup of flavonoids is flavanones such as hesperetin. Flavanones are abundant in citrus fruits like oranges, grapefruit, tangerines, lemons, and limes. In this respect, citrus fruits exhibit free radical scavenging characteristics and act as anti-inflammatory and blood lipid-lowering agents. Thus, the utilization of flavanones in the synthesis of multitarget-directed ligands (MTDL) has increased [97, 98].

#### 5.2.4. Chalcones

Chalcones are another significant subgroup of open chain flavonoids since the fundamental flavonoid skeleton structure lacks ring C. Numerous vegetables such as ladies' fingers and tomatoes contain a certain level of chalcones. Chalcones and their derivatives have attracted researchers' interest as anti-Alzheimer's agents due to their wide range of biological effects [97, 98, 102–105].

#### 5.2.5. Neoflavonoids

Neoflavonoids are natural products that belong to polyphenolic compounds. While flavonoids have the 2-phenylchromen-4-one backbone, neoflavonoids have the 4-phenylchromen backbone with no hydroxyl group substitution at position 2. In this respect, coumarin is a neoflavonoid found in various plants and has a wide range of therapeutic functions. According to molecular modeling studies, it interacts with AChE's peripheral anionic site (*PAS*) and parts as a potent inhibitor of AChE and inhibits A*β* aggregation [97, 98]. A derivative where the piperazine-based alkyl spacer connected both scaffolds to a new tacrine-coumarin hybrid was prepared. Due to its amide linkage, the product exhibited substantial inhibitory action against EeAChE (0.092 *μ*M) and moderate activity against EqBuChE (0.234 *μ*M), in addition to showing antiaggregation capabilities [106]. The 6 and 7 positions of coumarin are associated with alkyl spacers of various lengths with a terminal diethylamino group in coumarin-based MTDL derivatives, resulting in human AChE inhibition at nanomolar concentrations. In addition, these compounds exhibit remarkable inhibitory activity against A*β*42 self-aggregation (around 60%), thus providing a neuroprotective effect and making them a possible disease modifier [107].

### 5.3. Curcumin

Curcumin is a natural compound that has been used for centuries to treat a variety of diseases [108]. Curcumin's antioxidant and anti-inflammatory properties made it an effective neuroprotective drug to treat various neurological illnesses. Endoproteolytic breakdown of APP yields the A*β* peptide, which is 40-42 amino acids long. After combining A*β* with curcumin, research findings indicated that rats treated with A*β* show reduced oxidative stress, inflammation, and cognitive deficits [109, 110]. Curcumin's potential to prevent A*β* aggregation and fibril formation has been demonstrated in vivo and in vitro experiments. Along this line, amyloid plaques forming factors are metal chelation, low cholesterol levels, lipid peroxidation, facilitated transcription, and reduced production of *β*-secretase enzyme; curcumin modulated extracellular amyloid accumulation through these signaling pathways [111, 112]. In addition, curcumin suppresses protein aggregation by affecting the production of heat shock protein (HSP), which is another pathway. In this respect, HSP is molecular chaperones that prevent the formation of protein aggregation. Curcumin increased the formation of HSP in both in vivo and in vitro experimental settings. It also inhibited the formation of harmful amyloid aggregates and proinflammatory cytokines in the brain [111].

On the other hand, the intraneuronal tau protein's accumulations are another vital cause of AD. The *β*-sheet in tau protein, which drugs can inhibit like curcumin, causes aggregation. Moreover, curcumin's numerous systemic actions have made it a pleiotropic and cost-effective treatment for neuronal dysfunction [3]. Curcumin also suppressed the accumulation of A*β* in PC12 cells and human umbilical vein endothelial cells. In addition, curcumin exhibited antioxidant and anti-inflammatory properties in a Tg2576 mouse model of AD [113]. Similarly, curcumin exerted neuroprotective effects in primary neuronal cell culture against quinolinic acid-induced neurotoxicity. Findings showed that pretreatment with curcumin resulted in a substantial decrease in neuronal nitric oxide synthase [3].

### 5.4. Terpenes

Terpenoids are a collection of substances chemically known as 2-methyl-1 or 3-butadiene, generated biosynthetically from a mixture of two or more isoprene units [108]. *Tanacetum parthenium* contains parthenolide, a physiologically active sesquiterpene lactone that increases cognitive performance and lowers TNF-*α* and IL-6 levels in the cortex and rat hippocampus regions [114]. TLR4/NF-*κ*B-mediated reduction in the ranks of TNF-*α*, IL-6, and IL-17 in the brain ipsilateral hemispheres was recently shown to control neuroinflammation in intracerebral hemorrhage, which stimulates brain damage in rats [12]. Parthenolide's diverse inhibitory effects in multiple neuropathologies involving inflammation appear to be due to its NF-*κ*B inhibitory action. However, the neuroprotective effects of this sesquiterpene lactone must be validated in AD clinical studies.

Similarly, artemisinin, a sesquiterpene lactone present in the *Artemisia annua* of the Asteraceae family, was first used to treat multidrug-resistant malaria. This molecule and some of its synthetic counterparts have recently been found to exhibit promising neuroprotective action in AD, owing to their anti-inflammatory properties [115]. Because of their lipophilicity, artemisinin and its synthetic derivatives have been proven to pass the BBB [116]. Carnosic acid and carnosol, both present in *Rosmarinus officinalis*, are brain-permeable natural diterpenes with considerable neuroprotective activity [117]. No data demonstrate that ginkgolides are beneficial in AD treatment in clinical trials. Clinical trials involving *Ginkgo biloba* extracts resulted in mixed outcomes. They enhanced cognitive performance, neuropsychiatric symptoms, and functional capacities in patients with mild to moderate AD or vascular dementia, according to results of a randomized controlled trial (RCT) [12]. However, compared to placebo, another trial evaluated the efficacy of long-term use of *Ginkgo biloba* extract (120 mg) for reducing the incidence of AD in older persons with memory complaints; results showed that the extract does not diminish the progression of AD [118, 119].

### 5.5. Resveratrol

Resveratrol is an essential nonflavonoid found in red wine, almonds, and grapes [120]. Resveratrol exhibits numerous pharmacological activities, such as anti-inflammatory, antioxidant, anticarcinogenic, and antimutagenic [121]. It additionally demonstrated neuroprotective activity in vitro and in vivo models of AD. Aside from its antioxidant and anti-inflammatory properties, findings suggest that resveratrol enhances nonamyloidogenic APP division and aids in eliminating neurotoxic A*β* peptides, which is crucial in preventing and slowing down AD pathology [122]. Resveratrol also reduces neuronal cell loss through various mechanisms, the most important of which is the activity of NAD^+^-dependent histone deacetylase enzymes known as sirtuins [123]. Furthermore, resveratrol acts as an antioxidant by inhibiting ROS generation, increasing the amount of GSH and intracellular Ca21 in neurons, and altering the second messengers' calcium-dependent AMP-activated protein kinase (cAMP) and nitric oxide [124]. It also binds to A*β* plaques, which leads to the elimination of the A*β* peptide and inhibits AChE activity in in vitro cells [125].

## 6. Neuroprotective Mechanisms of Natural Products for AD

### 6.1. Antioxidative Neuroprotective Activity of Natural Products for AD

Reactive nitrogen species (RNS) and ROS are highly reactive molecules containing radical and nonradical oxidants. Processes such as cell cycle modulation, enzyme, and receptor stimulation, inflammation monitoring, phagocytosis, gene expression, and signal transduction depend on establishing a regulated proportion of these oxidizing agents in the human body [126–129]. In this respect, the nuclear factor E2-related factor 2 (Nrf2) route is one of the most used ways to regulate these reactive species. In response to OS, the transcription factor Nrf2 increases the production of antioxidant genes. The antioxidant response element (ARE) is intensely engaged in lowering OS, inflammation, and an assortment of a few toxic residues [130]. Since elevated metabolic activity and brain-limited cellular regeneration, OS has a more significant impact on the brain than other body regions [131]. Thus, OS is extensively regarded as a critical factor in gradually destroying neuron structure and decreasing neuron activity, one of the leading causes of NDDs, such as AD [127]. Therefore, much work has gone into developing antioxidant treatments as neuroprotective drugs for AD [132]. Published work showed that OS plays a vital role in advancing AD, and antioxidants act against OS damaging effects [133].

Natural and synthetic antioxidants are divided into two categories based on their natural prevalence, with the predominance of antioxidant compounds derived from natural sources. Vitamins A, C, and E, carotenoids, and flavonoids are the most well-known natural antioxidants, and they all contribute to protecting an organism from ROS-induced degradation. Numerous natural antioxidants, such as carotenoids and antioxidant vitamins, are derived from plants and correspond to phenolic and polyphenolic chemical structures, including hydroxyl groups on their aromatic ring(s). These natural compounds demonstrated substantial antioxidant activity as free radical scavengers and hydrogen atom donors [134]. Additionally, these phenolic and polyphenolics exhibit antioxidative functions due to their structures, notably the hydroxyl groups [135]. Flavonoids are the most abundant polyphenolic chemicals, and they possess a broad spectrum of antioxidative properties [136]. Furthermore, dietary polyphenols reduce neuron damage and apoptosis by lowering ROS levels [137]. Because phenolic compounds are naturally occurring substances, they reduce chronic NDDs, particularly AD. Polyphenolic compounds and their derivatives have recently received attention owing to their characteristics as neuroprotective agents for better management of AD [132].

### 6.2. Antineuroinflammatory and Neuroprotective Activity of Natural Products for AD

Although the specific pathophysiological process of AD has yet to be discovered, several assumptions such as the A*β*, the tau, the cholinergic, and the inflammatory hypotheses exist to interpret this complex disorder [138]. Along this line, the accumulation of A*β* in the brain, a prominent feature in the pathogenesis of AD, has been linked to neuroinflammation [139]. Elevated levels of ROS characterize the neuroinflammatory process in AD, enhance microglial activation, activate nuclear factor kappa B (NF-*κ*B), and increase cytokine production [140]. Additionally, immune cell activation leads to the secretion of IFN-*γ*, IL-1*β*, and TNF-*α* as proinflammatory cytokines [141] that drive neighboring astrocytes to produce A*β*42 oligomers [139]. These proinflammatory cytokines were discovered in high concentrations in AD patients' brains, serum, and cerebrospinal fluid [142]. In this respect, researchers have linked memory impairment in AD to elevated cytokine levels at all stages of the disease [143].

Moreover, it is associated with brain function, particularly in neurological diseases like AD. [144, 145]. Its activation has been associated with A*β*-initiated neurotoxicity [146], and it has been found in the brains of Alzheimer's patients [147]. Natural substances can hinder AD neurodegeneration with fewer adverse effects than synthetic medications. Additionally, natural compounds with anti-inflammatory effects may act as a pharmacological intervention to mitigate AD signs in the early stages [148]. The anti-inflammatory actions of these natural compounds are associated with several targets and several signaling pathways. In addition, natural products can stimulate the effects of antiamyloid as a sound output in AD management due to their ability to reduce neuroinflammation [149]. Consequently, natural substances or combinations of natural products should be evaluated for multitarget anti-inflammatory action as prospective therapeutic alternatives for managing AD [150]. Listed in Table 1 are neuroprotective of some plant-based natural products, extracts, and mixtures.

## 7. Therapeutic Targets for AD

### 7.1. Amyloid-*β* (A*β*) and Its Related Enzymes

Amyloid beta-peptide (A*β*) is obtained via the proteolytic processing of APP by *β*- and *γ*-secretases [226]. Published research demonstrated that immunization against A*β* has a neuroprotective effect and lessens memory deficits in transgenic mice without lowering the load of A*β* plaques [227, 228]. These findings show that A*β* in plaques may not cause synapse degradation, and that other types of A*β* play a significant role in neurotoxicity in AD brains [229]. Along this line, and based on evidence that A*β* plays a significant role in the etiology of AD, several therapeutic trials using passive and active vaccinations against A*β* were conducted [230]. However, neither technique was effective in clinical studies owing to side effects such as encephalitis and limited treatment efficiency. The action of *γ*-secretase and *β*-site *APP*-cleaving enzyme 1 (BACE1) on APP is required for the accumulation of A*β* peptide, which contributes to the pathophysiology of AD. Before its discovery, the BACE1 activity had been identified in cells and tissues [231].

On the other hand, overexpression of BACE1 enhances the synthesis of A*β* and BACE1-cleaved APP fragments [232]. In this respect, BACE1 splits APP carrying the Swedish familial AD causing mutation around ten to one hundred times more effectively than wild-type APP [231]. Moreover, some BACE1 inhibitors have been tested as antidrugs [233]. Alzheimer's early-onset AD is caused by mutations in presenilin genes [234]. Presenilins are multipass membrane proteins that were later discovered as *γ*-secretase catalytic components; the membrane implanted aspartyl protease complexes that generate the carboxyl terminus of A*β* from APP [235]. The *γ*-secretase activity is a therapeutic target for drugs that ease the amyloid plaque, whose accumulation is thought to cause AD.

Meanwhile, the notch signaling is associated with *γ*-secretase, a primary target for developing an AD therapy. In this regard, compounds that modulate A*β* synthesis by *β*-secretase without affecting Notch proteolysis and signaling are now in the therapeutic development pipeline at various levels. It is worth mentioning that flurbiprofen, a *γ*-secretase modulator, was the first drug to go through a clinical study. However, it was unsuccessful because of lack of therapeutic efficacy. However, numerous potent Notch-sparing inhibitors have recently been identified and tested in different clinical trial stages [236]; GSI-953 from Wyeth is an example [237].

### 7.2. Glycogen Synthase Kinase 3 (GSK3) and Tau Protein

Tau is a mature neuron's major microtubule-associated protein (MAP). It plays a vital role in the pathogenesis of AD and other related disorders, which are known as tauopathies, where tau is deposited in brain regions affected by the disease [238]. In the AD brain, tau is approximately three to fourfold more hyperphosphorylated than the average adult brain tau. Pathologic tau in AD is hyperphosphorylated and abnormally cleaved [239]. Accordingly, inhibition of abnormal hyperphosphorylation of tau offers a promising therapeutic target for AD and related tauopathies [228]. Recent studies have linked early modifications in the structures of soluble tau proteins, particularly their phosphorylation, to neurodegeneration [240, 241]. In this context, GSK3 is a potential kinase that regulates tau binding to microtubules, tau breakdown, and tau aggregation by phosphorylating tau protein [242]. Moreover, findings showed that A*β* enhances GSK3 activation and tau phosphorylation in AD [243]. Some GSK3 inhibitors are currently being studied for their therapeutic potential in AD. Furthermore, research findings revealed that AZD1080, a strong and selective GSK3 inhibitor, can inhibit tau phosphorylation in cells expressing human tau and in intact rat brain [244].

### 7.3. Acetylcholine-Related Molecules

Cholinergic deficiency owing to basal forebrain atrophy was detected in AD, in addition to the complicated clinical and metabolic abnormalities implicated in the neuronal symptoms of AD, such as the development of NFT and A*β* aggregation [17]. Furthermore, because of the favored loss of neurons expressing nAChRs, there is a significant drop in cerebral nAChR levels in AD [245]. Additionally, the interaction of acetylcholine and nicotinic ligands with nAChRs is essential in mental activities, and nAChR activation by nicotinic ligands can also protect neurons [246]. As a result, new chemicals have been developed to increase acetylcholine levels and directly stimulate nAChRs to reverse mental insufficiencies and shield neurons from A*β* neurotoxicity [247]. Thus, acetylcholinesterase (AChE) inhibitors that limit hydrolysis of acetylcholine and particular agonists for nAChRs have been discovered [248]. Since cholinergic deficiency is a persistent and early result of disease development, AChE has proven to be a critical treatment target for attaining clinical development in AD [249]. In this respect, galantamine, rivastigmine, and donepezil are three of the four medications now available for AD treatment. Galantamine, produced from the bulbs of the common snowdrop and various Amaryllidaceae, has been accepted for the symptomatic treatment of AD-associated senile dementia in several countries [250].

## 8. Discussion

AD is the most prevalent NDD, and it is one of society's major social and economic challenges. Due to the lack of specific diagnosis and treatment practices, AD is facing barriers in curative strategies. Several bioactive substances and natural extracts are mentioned herein to cure and prevent AD. Until today, most natural chemicals investigated have been sourced mainly from plant sources, with only a few molecules recovered from animals and marine sources. Because AD is a complex condition, these natural substances were linked to various treatment strategies. However, natural substances' neuroprotective effects are dependent on their capacity to penetrate the BBB.

Drug bioavailability and the difficulty of crossing the BBB are critical barriers to developing novel therapeutics. Both observational and experimental research show that bioactive substances enhance cognitive functioning in AD patients. Their modes of action vary, but the critical beneficial effects include the following: reduction of A*β* levels and tau phosphorylation rate, prevention of A*β* and tau aggregation, defense against OS, anti-inflammatory activity, and protection of cellular structures and inhibition of neuronal apoptosis.

Finally, despite recent significant discoveries, the development of potent and selective bioactive plant derivative natural compounds endowed with favorable ADMET (absorption, distribution, metabolism, excretion, and toxicity) properties, particularly higher metabolic stability and lower toxicity, remains a challenging goal for medicinal chemists. Thus, bioactive compounds will likely stay on stage for some time, even if no new natural compound has been advanced to the clinical trial stage in several decades. Compounds that have been shown to have neuroprotective properties in vivo should be investigated further in clinical studies.

## 9. Future Perspectives

In summary, AD is a terrible neurological condition that has affected humans for decades. Although few medications are accessible today for the treatment of AD, and several plants and their extracts have been widely used in animal studies and AD patients, no drug or plant extract could adequately reverse the symptoms of the disease [251–254]. AD is a multifactorial disease with various causes. Based on AChE inhibition or NMDA receptor antagonism, the present treatments provide some symptomatic relief but do not affect the disease morbidity or mortality. Because of these shortcomings, more serious work is required to comprehend the hallmarks of NDDs, their origins, and claimed therapeutic interventions. It is believed that combining natural product chemistry, medicinal chemistry, pharmacology, biology, and other related fields could be the most promising path to drug discovery and ensuring that natural products and natural-based products are more likely to become therapeutically relevant pharmaceuticals in the treatment and prevention of AD. The implementation of nanotechnology and nanocarrier-based techniques in the delivery of natural products and their separated constituents may aid in the improvement and enhancement of therapeutic responses and efficacy. The use of nanoparticles in the delivery system can improve the bioavailability of natural goods and their constituents.

## 10. Conclusions

Given the variety and complexity of genetic and epigenetic factors underlying AD, numerous abnormal physiological aspects such as exposure to environmental toxins and risk factors linked to cardiovascular abnormalities decreased quality of life diet, and aging should be considered in managing AD cases. In addition, more pragmatic and extensive quality control regulations protect the safety and potency of these neuroprotective agents. Furthermore, innovative techniques and methods to enhance CNS direct exposure of these neuroprotective agents, including implementation of nanoscience in the transfer of natural products, could occupy an essential role in stopping the progress of dementia. In short, this review has highlighted the ability of nature to prevent and treat AD. In this respect, fruits, spices, nuts, and herbs contain vital bioactive chemicals that can help prevent and treat various ailments, including AD, without serious adverse side effects.

## Figures and Tables

**Table 1:** Neuroprotective status of some plant-based natural products, extracts, and mixtures.

Plant	Extract	Neuroprotective outcomes	Study model	Reference
*Panax ginseng*	Root extracts	Reduced A*β* formation and aggregation, inhibited AChE, restored synaptophysin and ChAT activity, and decreased A*β* formation and aggregation	In vitro, in vivo	[151–157]
*Ginkgo biloba*	Leaf extract	Scavenged free radicals, averted the mitochondrial malfunction, activated the JNK and ERK pathways, and blocked neuronal death	In vivo	[158–160]
*Pistacia vera*	Kernel	Improved cognitive and motor deficits caused by inhibited cisplatin or vincristine	In vivo	[161]
*Phyllanthus emblica*	Ethanol extract	Improved learning, memory, and antioxidant potential; inhibited AChE activity	In vivo	[162]
*Hibiscus sabdariffa*	Anthocyanin-enriched extracts	Reduced memory impairment by decreasing STZ-induced neuroinflammation and amyloidogenesis	In vitro, In vivo	[163]
*Spirulina maxima*	Ethanol extract	Reduced hippocampus A*β*1–42, APP, and BACE1 expression levels, which reduced AChE activity; lowered hippocampal OS, and elevated BDNF levels	In vivo	[164–166]
*Ishige foliacea*	Phlorotannin-rich fraction	Lowered brain AChE activity, reduced OS, and activated the ERK-BDNF-CREB signaling pathway	In vivo	[167]
*Juglans regia*	Defatted protein	Lowered proinflammatory cytokine expression and AChE levels, extensively restored antioxidant enzyme levels and reduced NF-*κ*B expression	In vivo	[168–172]
Almond (*Prunus dulcis*)	Paste	Reduced AChE activity, lowered cholesterol and triglyceride levels, increased brain tryptophan monoamine levels and serotonergic turnover, and improved learning and memory.	In vivo	[173–176]
Hazelnut (*Corylus avellana*)	Kernel	Improved memory, reduced anxiety, and lowered neuroinflammation and apoptosis	In vivo	[173, 177, 178]
*Vitis vinifera*	Juice, polyphenolic extract	Exhibited antioxidant, antineuroinflammatory, and antiamnesic properties and inhibited A*β* aggregation	In vivo	[179–184]
*Oryza sativa*	Dietary supplement	Reduced hippocampal AChE activity and lipid peroxidation products	In vivo	[185]
*Zingiber officinale*	Root extract	Acted as AChE inhibitor, suppressed lipid peroxidation, caused NMDA receptor overstimulation, and inhibited the generation of free radicals	In vivo	[186, 187]
*Benincasa hispida*	Aqueous extract	Prevented substance P (SP) formation, as were antioxidant scavenging effects.	In vivo	[188]
Fuzhisan	Herbal complex	Exhibited antiapoptosis and anti-A*β* buildup activity, increased ACh concentrations, and provided neurotrophic benefits	In vivo	[189–192]
Bojungikgi-tang	Herbal formula	Prevented the accumulation and A*β* peptide expression, NeuN, and BDNF in the hippocampus by inhibiting the aggregation of A*β*, enhanced BACE activity in vivo, and increased antioxidant action	In vitro, in vivo	[193]
*Pistacia integerrima*	Gall extracts	Exhibited cholinesterase inhibitory and free radical scavenging activity	In vitro	[194]
*Phyllanthus acidus*	Methanol extract	Increased brain antioxidant enzymes, improved cognitive functioning, and reduced OS	In vitro	[195]
*Hedera nepalensis*	Crude extract	Increased catalase (CAT) and superoxide dismutase (SOD) levels and decreased glutathione (GSH) levels	In vivo	[196]
*Thalassospira profundimaris*	Crude extract	Preserved the synaptic structure and prevented cell cycle-related neuron death	In vitro, in vivo	[197]
*Eisenia bicycles*	Methanol extract	Attenuated OS and reduced neuronal cell death	In vitro	[198]
*Curcuma longa*	Ethanol extract	Reduced CeCl3-induced OS, increased antioxidant enzyme activity, and inhibited AChE activity	In vitro, in vivo	[108, 199–205]
*Allium sativum*	Aged garlic extract	Reduced microglial activation and IL-1 levels and the inflammatory response and reduced psychological stress via modulating stress hormones and the OS response in the brain	In vivo	[206–209]
*Momordica charanti*	Dried and ground fruit	Reduced gliosis, oligomeric A*β* levels, tau hyperphosphorylation, and neuronal death; increased synaptic-related protein and pS9-GSK3b expression levels	In vitro, in vivo	[188, 210]
*Bacopa monnieri*	Extract	Reduced cholinergic degeneration, improved cognition, and suppressed AChE activity	In vivo	[211–216]
*Viscum album*	Extract	Significantly raised serum BDNF levels and reduced AlCl3-induced neurotoxicity	In vitro, in vivo	[217]
*Pistacia atlantica*	Ethyl acetate and aqueous extracts	Inhibitory action of AChE	In vitro	[218]
*Nardostachys jatamansi*	Ethanol extract	Inhibited cell death caused by A*β*	In vitro, in vivo	[219, 220]
*Phyllanthus amarus*, *Cynodon dactylon*	Methanol extract	Increased superoxide dismutase, catalase, and NADH dehydrogenase levels	In vivo	[221]
*Salvia miltiorrhiza*	Root extract	Inhibited OS and the mitochondria-dependent apoptotic pathway, inhibited production of NO and iNOS expression, induced neuron cell development in rat mesenchymal stem cells, and enhanced the differentiation ability of iPSCs and the survival and neuronal maturation of iPSC-derived neurons transplanted	In vitro, in vivo	[222–225]
